# The Medium Is the Message: How Do Canadian University Students Want Digital Medication Information?

**DOI:** 10.3390/life10120339

**Published:** 2020-12-10

**Authors:** Helen Monkman, Andre Kushniruk, Elizabeth Borycki, Debra Sheets, Jeff Barnett, Christian Nøhr

**Affiliations:** 1 School of Health Information Science, University of Victoria, Victoria, BC V8P 5C2, Canada; andrek@uvic.ca (A.K.); emb@uvic.ca (E.B.); jebarnet@uvic.ca (J.B.); 2 School of Nursing, University of Victoria, Victoria, BC V8P 5C2, Canada; dsheets@uvic.ca; 3 The Maersk Mc-Kinney Moller Institute, University of Southern Denmark, 5230 Odense, Denmark; cn@mmmi.sdu.dk

**Keywords:** consumer health informatics, consumer medication information, patient medication information, patient information leaflets, online medication information, user needs, mhealth

## Abstract

(1) Background: To facilitate optimal prescription medication benefits and safety, it is important that people are informed about their prescription medications. As we shift towards using the digital medium to communicate medication information, it is important to address the needs and preferences of different user groups so that they are more likely to read and use this information. In this study, we examined what digital medication information (DMI) format Canadian University students want and why. (2) Methods: This study was a qualitative investigation of young (aged 18–35) Canadian University students’ (*N* = 36) preferences and rationale supporting these preferences with respect to three potential formats for providing DMI: email, a mobile application (app), and online. Reported advantages and disadvantages of each of the three DMI formats were identified and categorized into unique themes. (3) Results: Findings from this study suggest that Canadian University Students most want to receive DMI by email, followed by a mobile app, and finally they were least receptive to online DMI. Participants provided diverse themes of reasons supporting their preferences. (4) Conclusions: Different user groups may have different needs with respect to receiving DMI. The themes from this study suggest that using a formative evaluation framework for assessing different DMI formats may be useful in future research. Email may be the best way to share DMI with younger, generally healthy, Canadian University students who are on few medications. Further research is required to explore whether other mediums for DMI are more appropriate for users with other characteristics (e.g., older and less educated) and contexts (e.g., polypharmacy and complex conditions). Given the flexibility of digital information, DMI could plausibly be provided in multiple formats and could allow users to choose the option they like best and would be most likely to use.

## 1. Introduction

“The medium is the message.”

~Marshall McLuhan

This quote is often interpreted to suggest that emphasis should be placed on the medium by which the message is communicated, rather than the content of the message itself [1]. Arguably, the medium should not be the sole focus of communication, nor should its importance be ignored [1]. To enhance our understanding of how Canadian University students prefer to receive and access information about their prescription medications, we used qualitative methods to investigate digital format options for medication information, but not their content or design. Specifically, we explored peoples’ preferences about the following formats of digital medication information (DMI) delivery: email, online, and mobile applications (apps) as well as their rationale behind wanting to receive it that way.

### 1.1. Background

Prescription medication use is common globally and per capita pharmaceutical spending is trending upward internationally [2]. Taking any medication has inherent risks and benefits. To increase safety and appropriate use of taking prescription medications, people need to be informed. For example, people need to know (a) whether they feel comfortable taking the medication given its associated risks and benefits (b) how to administer the medication, and (c) how to recognize and respond if side effects or allergic reactions occur. There are several different ways medication information about prescriptions is communicated to people. For example, people may receive verbal counselling from their prescribers (e.g., doctors, nurse practitioners) when the prescription is written as well as from pharmacists when the prescription is picked up. Where necessary, warning labels are affixed to the medication packages themselves to highlight important aspects related to medication taking behaviour. In addition, there are several forms of prescription information meant for citizens which encompassed in the term Written Medication Information (WMI). Examples of WMI include patient package inserts, consumer medication information, patient information leaflets, and medication guides. Some WMI is written by the pharmaceutical manufacturer (e.g., patient package inserts) and other WMI is written by independent organizations or a pharmacy chains (e.g., consumer medication information). However, the goal of all WMI is to facilitate understanding of how medications should be taken safely and effectively [3].

The names and regulations for WMI vary internationally [3] and the digital formats different countries use to provide this information to its citizens vary as well. For example, Canada is still primarily a paper-based practice. With a new prescription, Canadians receive patient medication information (PMI; formerly consumer medication information) printed on paper at the pharmacy. PMI is a specific type of WMI that is written by organizations other than the manufacturer and is not regulated by the federal government, but ought to align with the product monograph, which is regulated by Health Canada [4]. Thus, the printed information Canadian citizens receive about new prescriptions can vary between pharmacies for the same medications [5]. Moreover, different topics are discussed or omitted in patient medication information depending on the particular medication. For example, there are no administration instructions for inhalers, instead people are redirected to patient package insert [6]. In addition to PMI, citizens may also receive a patient package insert and/or stickers on the package as well. Discrepancies, redundancies, and failure to address user needs and capabilities in different types of American WMI have also been observed [7]. As a result “the FDA [US Food and Drug Administration) sees merit in adopting a single, standardized PMI [patient medication information] document to accompany dispensed prescriptions” (p. 162) that would be made publicly available online [7]. Subsequent progress has been made in developing the format and guidelines for new and improved materials (e.g., [8,9]).

### 1.2. International Approaches to Digital Medication Information

As with many other types of information, information about prescription medications is also beginning to be offered digitally. We define digital medication information (DMI) as information offered electronically about prescription medications to support citizens in taking these medications safely, properly, and efficaciously. DMI could be offered in various formats such as emails, websites, or mobile applications.

Countries have taken different approaches to deploying DMI and are using hybrid (i.e., paper and digital) methods to share information about prescription medications with their citizens. For example, some Canadian organizations are beginning to deploy DMI as a complement, but not replacement for WMI. For example, some Canadian pharmacies also offer medication information on their websites. However, this information is not necessarily consistent with what people receive on paper from the same pharmacy chain [5]. Further, many websites developed by pharmacies or other agencies with medication information targeted to citizens are often not designed to be used and understood by people with limited eHealth literacy [10]. Recently, some Canadian pharmacies have also introduced mobile apps to facilitate prescription management and ordering refills. These apps may offer information about prescriptions such as an image of what the medication looks like, when it was last filled, amount or number remaining, when it expires, identification numbers. These apps also may include features such as accessing family member’s prescription profiles, making appointments for medication reviews, and scheduling pickup or delivery of medications. Additionally, some of these apps also offer the same patient medication information that is dispensed on paper at the pharmacy [11]. However, the adoption rate of these prescription management apps is unknown. Although DMI is beginning to emerge in Canada, informing citizens about prescription medications is still primarily a paper-based process.

Generally, there is a dearth of literature regarding international approaches to informing citizens about prescription medications. Moreover, these approaches are not typically reported in the grey literature either. Instead, informing citizens about prescription medications tends to be a practice that is done internationally, but not fully elucidated and must either be determined by insinuation or by asking people of different nationalities to explain the practices of their countries. Given these challenges, we have compiled a few international examples based on what was identified in the literature, and by communicating with international colleagues.

Similar to Canada, the United States and Sweden appear to have hybrid but primarily still paper-based approaches to informing their citizens about new prescription medications they are taking. As in Canada, the United States offers paper leaflets to accompany new prescriptions at the pharmacy [12]. However, as in Canada, some American pharmacies also offer medication information on their websites [10] and citizens can also review medication guides and are encouraged to the website by the National Institutes of Health, MedlinePlus: Drugs for prescription medication information [13]. Recently, some authors have argued for the need to update guidance for consumer medication information to include considerations for delivering this information via mobile devices [14]. Similar to Canada and the United States, Sweden also appears to be using a hybrid approach to providing its citizens prescription medication information, with one significant difference: Sweden provides printed patient information leaflets (i.e., a type of WMI), complemented with single national website for medication information www.fasse.se to complement the paper print outs [15]. Similarly, Australia offers consumer medicine information (a) on a website called Medicine Finder (nps.org.au/medicine-finder); (b) by having doctors and pharmacists print it for citizens; (c) within some packages; (d) by phone; (e) from the pharmaceutical company [16] and through a complementary medication management mobile app [17]. Australia’s multipronged approach drawing from a single repository is what the United States is working towards [7]

Thus, Sweden and Australia’s approach differs from Canada and the United States whose individual pharmacies and some regulatory bodies offer online medication information rather than having a single national resource.

In contrast to these hybrid approaches, still relying primarily on WMI and in the early stages of DMI deployment, some European countries have implemented strictly digital strategies to convey information about prescription medications to their citizens. For example, Finland offers medication information to its citizens using a website called Lääkeinfo (translation: Drug Information; laakeinfo.fi). Denmark is another key example of a country that has adopted a more digital strategy to inform its citizens about prescription medications. Approximately 5.8 million people live in Denmark [18]. A Danish organization called Medicin.dk has been publishing information about medications since 1975 and provides information about medications to all Danes [19]. Medicin.dk’s vision is to “make a valuable contribution to Danish society by giving health professionals and citizens easy and equal access to credible, in-depth and useful knowledge about prevention and treatment with medicines”. To this end, Medicin.dk currently offers three integrated websites: (1) Pro.medicin a source targeted to health professionals, (2) Min.Medicin (translation: My Medicine) a resource targeted to citizens, and (3) Indlaegssedler (translation: Patient Package Inserts) another resource for citizens [19]. All prescription medications have information on Min.Medicin and the information on Min.Medicin is most similar to Canada’s PMI. Some packaged medications (e.g., come in a box) also include additional information printed on paper included in the package called patient package inserts. Indlaegssedler contains the same information as the patient package inserts. By offering paper package inserts digitally, Indlaegssedler aims to increase the readability of patient package inserts over the printed versions by using larger font, being more spacious, and not having text obscured by folds in the pages [20]. In May 2020, there were approximately 693,000 visits to Min.Medicin and 1,650,000 pages were viewed, which equates to slightly more than 2 pages per visit [21]. In the same month, there were approximately 154,000 visits and 264,000 pages viewed on Indlaegssedler, which is equal to slightly less than 2 pages per visit [21]. Thus, Min.Medicin is used approximately 4.5 times more frequently than Indlaegssedler. Higher usage of Min.Medicin may attributable to the fact that Danes also receive the paper package inserts with redundant information to Indlaegssedler. Additionally, as previously described not all medications have paper package inserts, so there is a smaller sample of medications represented on this site.

DMI may have several potential advantages over WMI. For example, both Min.Medicin and Indlaegssedler have collapsible content and multimedia (e.g., pictures, videos). Digital information also makes it possible for people to adjust the font size and offers layered content with progressively more detail. In addition, other posited benefits of DMI include ensuring the information is current, additional usability (e.g., definitions of medical terminology) and accessibility features (e.g., text to speech) [15]. Although some websites that provide online medication information for citizens, many sites fail to leverage the possibilities of this format over what is possible on paper, many do not [10,22]. The paper medium is a reported deterrent for WMI use because in many instances it is unavailable when people might need to use it [23]. To that end, DMI has the advantage of being readily accessible than paper WMI. Thus, DMI has multiple potential benefits over the paper medium.

Despite the increase in DMI options available, it is not prudent to assume that people prefer receiving DMI over traditional paper based WMI. Moreover, there is scant research devoted to the preferences of individuals regarding DMI. Instead, it appears that DMI deployment strategies have not been informed by evidence regarding how people would like to receive and use DMI, but rather followed the trend of adopting digital means of communication over paper methods. In addition to our work, we only identified a single study exploring preferences around DMI. Specifically, a Swedish study (*N* = 406) found that 52% of their respondents preferred the paper leaflets and only 17% would prefer to read the leaflets on a computer, phone, or tablet (i.e., DMI) instead [15]. However, the aforementioned study posed the question to participants generally, as general digital alternative, but failed to explore specific means for how DMI might be provided to citizens. Although digital was not the preferred format, 41% of respondents still felt positive towards DMI and this proportion was even greater for those younger and up to 55 years old [15]. Similarly, in a another branch of this study, using the same sample of Canadian University Students as the current study, most of these participants (*N* = 36) reported they would rather receive prescription medication information digitally than printed on paper [24]. However, many participants also argued that WMI should still be a choice for other citizens who might prefer it on paper [24]. Arguably, peoples’ preferences regarding DMI may affect not only how receptive they are to it, but also their likelihood of using it.

### 1.3. Research Questions

As previously described, currently, the most common form of DMI internationally seems to be national websites, yet there are alternatives for delivering DMI that are emerging and/or worthwhile exploring (e.g., mobile apps, email). These three possible DMI methods (i.e., websites, mobile apps, email) may elicit different opinions from users and different options may be more suitable for users depending on their preferences and specific needs. Therefore, in this qualitative study, we explored Canadian University students’ preferences, as well as the reasons behind these preferences, about possible formats of digital media for receiving information about their prescription medications (i.e., DMI). That is, we sought to answer the following research questions:

1. What format do young adults want as a DMI format (i.e., online, mobile app, or email)?
2. Why do they have that DMI format preference?

## 2. Materials and Methods

### 2.1. Participants and Recruitment

The Human Research Ethics Board at the University of Victoria approved this study (ethics protocol number 16-198). We recruited participants using posters distributed around the University of Victoria campus and by emailing the School of Health Information Science’s undergraduate listserv. Participants were eligible to participate if they were over 18 years of age, had corrected to normal vision and hearing, and were neither health care professionals nor pursuing a health care professional degree.

### 2.2. Setting and Context

To participate in this experiment, participants came to the principal investigator’s (HM’s) office on the University of Victoria campus in Victoria, British Columbia, Canada. The questions posed in the current study were asked within the scope of a broader experiment exploring potential differences in memory and perceptions of multimedia medication information for citizens (see Monkman, 2018). The entire experiment was approximately an hour in duration per participant.

### 2.3. Procedure

This study focusses on two questions posed to participants as part of a broader experiment [24] and other findings have already been published [23,25]. The broader experiment began with participants using the University of Victoria’s instance of SurveyMonkey^®^ to respond to a demographic questionnaire, the Newest Vital Sign (NVS; a health literacy scale) [26], and the eHealth Literacy Scale (eHEALS) [27]. Next, SurveyMonkey was also used to explore participants’ memory, perceptions, and preferences for different examples of multimedia consumer medication information formats [25]. Finally, the experiment concluded with semi-structured interviews investigating participants’ perceptions and lived experiences relating to the Canadian prescription process, as well as how and when they were informed about prescription medications [24]. During the semi-structured interview of the broader experiment, we posed the following two questions, which will be the focus of this study:

1. If you were to receive digital medication information, how would you like to receive it? For example, online (website), email, or a mobile app?
2. Why?

Notably, we asked these questions without providing participants any guidance about what the different formats might contain or what features they might have. Thus, the participants generated their own ideas of how DMI formats would look and behave. Participants’ responses to these questions were audio recorded.

### 2.4. Analysis

Descriptive statistics were used to summarize the characteristics of the sample. The semi-structured interview recordings were transcribed and imported into MAXQDA^®^ for analysis. We analyzed participants’ responses to these questions using directed and conventional content analysis to capture both existing and emergent themes respectively [28]. First, we analyzed the transcripts to identify which of three DMI categories each participant indicated they would prefer to receive. When identified two as being equally suitable alternatives rather than selecting a single preferred DMI format, 0.5 was allocated to each of the two preferred formats. To guide the directed content analysis [28], we developed a simple coding scheme to categorize participants’ quotes that related to the questions we posed be began by using a simple two tiered hierarchical structure. First, quotes were categorized based on what DMI format they referred to (i.e., online, email, mobile app) and then within each format category whether or not they represented either an advantage or disadvantage of that format. To quantify these results, we used the sum of participants who reported at least one (but could have named more) advantages and disadvantages for each format. That is participants were only counted once per advantage or disadvantage in each DMI format, regardless of how many positive or negative comments they made about that format. Subsequently, we wanted to understand reasons why participants believed one format was more suitable than another. To this end, we used conventional content analysis [28] to identify emergent themes examining all of the quotes (i.e., advantages and disadvantages of all three DMI formats) for commonalities that described why they believed one format was better or worse than another. In some comments, participants described disadvantages of one DMI format as the rationale supporting another format. To summarize this data, we identified and defined all of the common themes and for each DMI format noted which themes were discussed by participants and whether they were regarded as advantageous or disadvantageous, or both.

## 3. Results

### 3.1. Demographic Data

A total of 36 young adult (i.e., 35 years old or younger) Canadian University students participated in this study. Participants (*N* = 36) were on average 23.6 years old (*SD* = 3.8, range = 18–35). We did not have an a priori upper age limit on people who could participate. However, to prevent possible heterogeneity in perceptions due age differences, we excluded two participants from this analysis because they were substantially older than the rest of the sample (i.e., >3 *SD* from the mean age). This sample (*N* = 36) of Canadian University students was predominantly female (26, 72.2%) and identified as Caucasian (23, 63.9%) or Chinese (6, 17.7%). The majority spoke English as their first language (31, 86.1%) and felt very comfortable using computers (30, 83.3%). Most (30, 83.3%) felt that they grew up in working class families and half of the sample (18, 50.0%) currently worked either part time or full time themselves.

All of the participants were currently enrolled in either full-time (30, 83.3%) or part-time (3, 8.3%) studies or were on co-operative education work terms (3, 8.3%). Half of the sample (18, 50.0%) reported high school as the highest level of education completed and the other half had either previously completed an undergraduate degree (13, 36.1%), or graduate degree (4, 11.1%). Only one participant (2.8%) indicated other. Participants were pursuing their degrees in the faculties of Science (9, 25.0%), Social Sciences (8, 22.2%), Human and Social Development (7, 19.4%), and Education (4, 11.1%), with the remaining 8 (22.2%) participants belonging to other faculties.

Participants turned to a variety of resources for medication information and often reported relying on more than one resource for this type of information. Reported resources for medication information included physicians (27, 75%), pharmacists (16, 44.4%), electronic resources such as the internet (16, 44.4%), or family members (9, 25%). Although many participants (16, 44.4%) took no prescription medications, the majority took either one (13, 36.1%) two (16.7%) or three (1, 2.8%). Scores on the Newest Vital Sign (NVS) [26] revealed that there was a low likelihood that most participants (30, 83.3%) had limited health literacy and the remaining six (16.7%) only possibly had limited health literacy. However, these results were contrasted by a larger distribution of scores on the eHealth Literacy Scale (eHEALS), which motivated a discussion about the relationship, or lack thereof, between health literacy and eHealth literacy [29].

### 3.2. DMI Data: Which DMI Format Did Participants Prefer?

**Figure 1:**
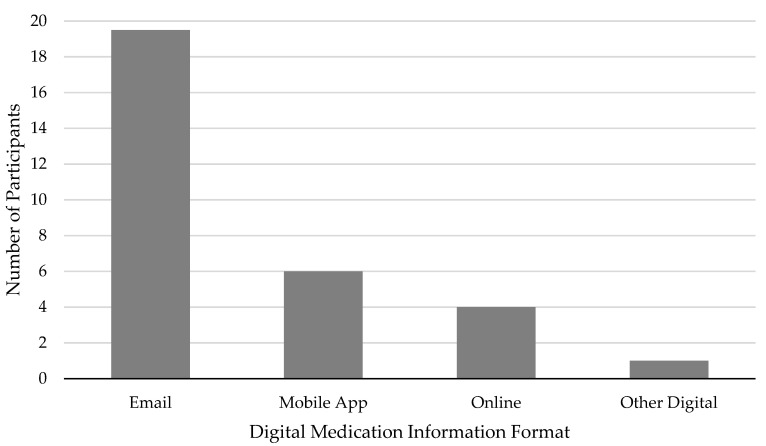
Number of participants who preferred different digital formats. Note: Participants who discussed 2 preferred options were counted as 0.5 for both.

Participants’ overall preferences of the three proposed methods of DMI distribution (i.e., email, online, mobile application) were examined and patterns emerged. As previously reported, this sample generally preferred DMI over WMI, but many of them also asserted that people should have the option about how they receive prescription medication in print and/or digitally [30]. In this more thorough investigation of preferences towards three types of DMI formats, participants were most receptive to email (19.5, 54.2%), followed by a mobile app (6, 16.7%), and finally online DMI (4, 11.1%; see Figure 1). One participant (2.8%) suggested distributing DMI to people before and ensuring they used it before they would be allowed to pick up their prescription at the pharmacy (see Figure 1). Specifically, Participant 36 said: “I think that would get people to actually know how to take things instead of—because a lot of people just disregard them.” Interestingly, 5.5 participants (15.3%) were not receptive to DMI and maintained that they prefer the current paper process. However, given that we sought to compare and contrast amongst DMI formats and not between digital and paper medium, these responses were excluded from further analysis.

### 3.3. DMI Data: Why Do Participants Believe Email is Most Suitable DMI Format?

**Figure 2:**
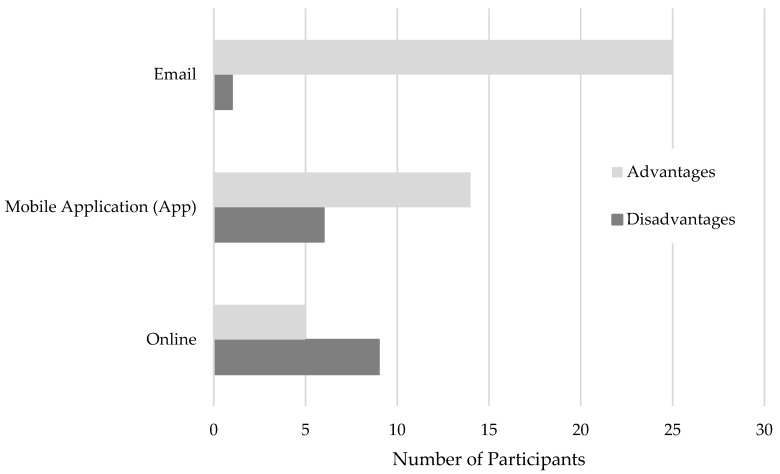
Number of participants who reported of advantages and disadvantages for each format of digital medication information (DMI). Note: Total number of participants exceeds the sample size because they could report advantages for more than one DMI. However, participants were only counted once per format regardless of how many advantages they reported for that format.

Intuitively, the pattern of the results for advantages and disadvantages of the three formats paralleled the preferences data. Specifically, the most participants reported at least one advantage of email, followed by mobile app, and finally online (see Figure 2). Additionally, there was an inverse relationship with the number of participants who reported at least one disadvantage of each of the three DMI formats, although fewer participants overall reported disadvantages of any format (see Figure 2).

To further investigate the rationale for why people preferred one DMI format over another, we examined the distribution of advantages and disadvantages reported for each format in the different themes. In total, we identified the following 15 unique themes of DMI based on participants’ comments:

1. Availability: Can be used at multiple locations;
2. Ease of Access: Does not require and account or login and password;
3. Findability: Easy to locate;
4. Communication Method: Sent to users, sought out by users, or allows people to interact in real time;
5. Storage: Easy to keep, requires limited storage space;
6. Security: Ensuring one’s prescribed medications are kept secure and confidential;
7. Flexibility: Offers different ways of sharing, storing, or using the information;
8. Personalization: Tailored to an individual’s situation;
9. Searchability: Can search the document for specific information;
10. Frequency of Use: How often people would need to refer to the information;
11. Trustworthiness: Comes from a reputable source;
12. Comprehensiveness: Contains all prescription medication related information in one place;
13. Layered Content: Provides different levels of detail;
14. Helpful in an Emergency: Provides access to prescribed medications to first responders;
15. Medication Reminders: Provides notifications to support medication adherence.

The pattern of themes as advantages and disadvantages associated with each of the three DMI formats was unique. Specifically, participants’ comments related to the advantages of email for DMI belonged to 10 themes, compared to seven for a mobile app, and six for email (see Table 1). In contrast, there were two disadvantages discussed by participants regarding email for DMI, three for a mobile app, and three for online (see Table 1). Thus, in contrast to the range of unique themes for advantages of DMI, there were fewer themes of disadvantages identified.

Each of these themes was based on one or more quote made by participant(s) and therefore can be illustrated by representative quotes. To see participants quotes that provide examples of these themes and whether or not they were considered advantages or disadvantages, see Table 2 for email, Table 3 for mobile app, and finally Table 4 for online.

## 4. Discussion

The advantages and disadvantages varied amongst DMI formats varied. However, all three options (i.e., email, mobile app, and online) offered increased availability. Although all DMI formats increase availability, results from this study suggest that generally, younger, healthy, Canadian University students would like to have their prescription medication emailed to them. Participants generated a number of unique reasons why email suited them best and why other options were not appropriate. Firstly, they considered email easy to access because it did not require an additional login (accessibility) and made it easy to locate (findability). They wanted to receive the DMI rather than have to go looking for it (communication method). Participants did not want something that would require storage space on their devices (storage space), would require logging in to access the DMI and wanted it to be easy to access. However, email DMI was also considered less secure, which was a drawback for some. There is an inherent tradeoff between accessibility (i.e., no login) and the security of the information. Many participants appeared to prioritize the ease of accessing DMI over having it kept private and secure. However, this requires further investigation.

**Figure 3:**
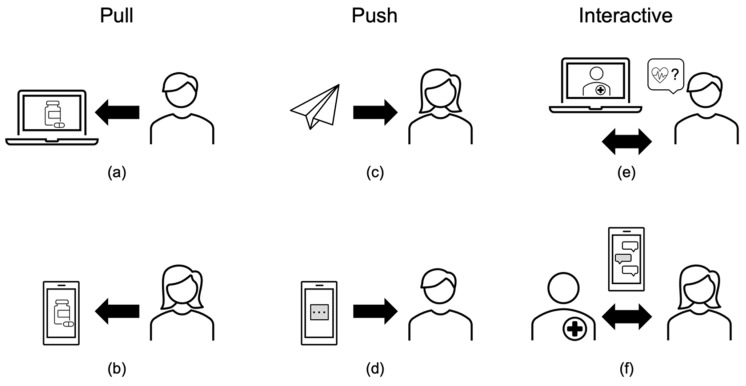
Examples of digital medication information (DMI) using three different communication methods: (**a**) pull communication, person seeking online DMI; (**b**) pull communication, person seeking DMI using a mobile app; (**c**) push communication, DMI emailed to a person; (**d**) push communication, a notification from a mobile app that DMI is available; (**e**) interactive communication, a person asking a pharmacist questions virtually; (**f**) interactive communication, a person asking questions to a pharmacist using by texting in a mobile app.

Findings from this study suggest that people prefer push communication to pull communication for DMI. Three types of communication methods are pull, push, and interactive [31] which can be exhibited in potential DMI (see Figure 3). Pull communication requires recipients to seek out and retrieve information of their own volition [31]. In contrast, push communication refers to information that is sent to people (i.e., recipients) who may not have requested it [31]. Both push and pull communications methods are unidirectional in that the information only flows one way. In contrast, interactive communication is a real time, back and forth exchange of information and is considered the most effective way of ensuring understanding [31]. Examples of the pull communication method for DMI are websites such as www.min.medicin.dk. and www.fasse.se which citizens must seek out. Alternatively, if people went into a mobile app to get their DMI, that would be another example of pull communication. However, instead of putting the onus on citizens to retrieve the DMI, push communication for DMI would be sending it to people by email or notifying them that it is available through a mobile app. Finally, examples of the interactive communication method in DMI would be having a pharmacist available to chat (either using messaging or virtually) with people about their new prescriptions either online or using a mobile app. Previous research has shown that people would appreciate the opportunity of having convenient access to follow up care to support prescription medication use [30]. For illustrated examples of all of the aforementioned communication methods applied to DMI, see Figure 3. Thus, we can see different DMI formats can utilize one or more communication methods and these communication methods may also impact peoples’ opinions and use of DMI. Ideally, we want people to use DMI so that they are informed of a prescription medication’s associated risks and aware of how to maximize its benefits.

Participants in this study recognized that user needs may influence which DMI format is considered most ideal and that their opinions might not be representative of other types of citizens. That is, this sample described that their need for a mobile app was limited because they had few medications, no complex health conditions, and/or would use it infrequently. However, they recognized that a mobile app may be more suitable for people with different needs and characteristics. Additionally, five participants preferred paper over any type of DMI and in previous work participants reported that offering both print and digital medication information would be most ideal so that citizens have the choice of which format suits them best [24].

Interestingly, one participant noted that people often disregard medication information entirely and suggested it should be mandatory to review before receiving the prescription medication. Although this approach might increase the likelihood of people using DMI overall, it limits the availability of DMI for future reference, people may not be receptive to this approach.

In this study, we did not define what we meant by any of the suggested DMI formats nor did we propose any associated features. We wanted participants to envision how they would like to receive DMI rather than having them bound by what was or was not possible and/or currently available. This approach did create some variation in participants’ responses because they held their own ideas which were potentially inconsistent amongst participants. For example, emailed DMI could contain all of the information in the body of the email, a link to a website, or a PDF of the information. Additionally, some believed online DMI would be a password protected, secure database and others imagined it as a generic website with open-access repository. Although participants had different ideas about the specifics of what features DMI would offer, we still garnered insight into a variety themes that should be examined moving forward (see Table 1). This opportunity for creative freedom about how DMI would work also resulted in participants generating ideas about how DMI could be conveyed most effectively in terms of its content (e.g., personalized, layered content), combined with other information about prescription medications (e.g., comprehensiveness), and what potential features (e.g., emergency access to current prescriptions, reminders to take medications) could be included in DMI. Additionally, the vagueness in DMI descriptions was beneficial in this exploratory phase of research, as it allowed creativity and idealized DMI solutions for each of the three DMI formats.

Findings from this study suggest that although efforts are being made to digitize medication information for citizens, their chosen formats may not align with user preferences or at least Canadians University students’ preferences. For example, many countries offer DMI on national portals. However, by having people seek out (i.e., pull communication method) DMI there are two potential issues: (1) people do not go to the intended trustworthy site; (2) people just avoid getting any information at all. Participants in this study described not only the inconvenience of having to seek out online DMI, but also having to remember what it is called or how to get to it. Moreover, how likely are citizens to use a website from national agency or pharmacy compared to other online medication information resources? Theoretically, by requiring citizens to search for a resource, alternatives are generated and therefore users may be more likely to use another site than the one they originally sought out.

Australia’s multipronged strategy may satisfy the most peoples’ needs by offering medication information in print, on a website, in an app, and by phone. However, all of possibilities to access medication information are pull communication methods. DMI is not emailed to Australians. In fact, we were unable to find any existing examples of emailed DMI. Therefore, we may not be capitalizing on an opportunity to increase the use of medication information by using a push method of communication.

The internet has been an important resource for information on prescription medications [32] and people have been using it for this purpose for many years [33]. However, early research suggested that people tended to use search engines to find the information and were not adept at appraising these websites nor did they have insight to their approach of information seeking [32]. It is unclear whether these same traits are present in people using the internet for information about prescription medications today. Recent research suggests that online medication information for citizens is often not well designed and communicated, which can be especially problematic for people with limited eHealth literacy [10]. The results of this study suggest that there may be many benefits to providing DMI by email and this might be an inexpensive way to offer DMI and ensure that people at least receive it. Further, as is common practice in retail stores, people could have the option of having the information printed for them or receive it by email [30]. Moreover, as pharmacies begin to deploy mobile apps, it is important to consider the features people want to support safe and effective medication taking (e.g., reminders, access to health care professionals). These apps could also serve to centralize everything to do with one’s prescriptions (e.g., refills remaining) and facilitate medication management while eliminating what is referred to as information scatter [34].

The primary limitation of this study is the sample size and its characteristics which potentially limit the generalizability of these results. Specifically, there were only 36 participants and generally they were healthy, young adult Canadians, with adequate health literacy, working while pursuing post-secondary education who came from working class families. Thus, the generalizability of these results is limited, as the opinions of this sample may not be representative of people who are non-Canadians, have chronic conditions, have lower levels of health or eHealth literacy, are older, or less educated. Additionally, using the word “receive” in the interview question may have primed participants to respond more positively to push communication methods. However, this phrasing is consistent with the current paper practice which uses the push communication method with paper information. A term such as obtain or get may be less likely to influence participant responses and be more appropriate in other national contexts whereby citizens have to seek out this information (e.g., Denmark). Moreover, one of our recruitment methods was email, which may have resulted in our sample being more biased to email communication. Despite the limitations of this study, several interesting findings were revealed that warrant further exploration.

There are several future directions for this work. Firstly, future research should examine whether preferences and needs are stable or vary between different user groups. Secondly, peoples’ opinions of existing DMI options and prototypes should be investigated. Thirdly, the findings from this study could be used as a framework and the relative importance of the themes revealed in this study should be explored. Fourthly, it would be worthwhile to investigate the extent of the influence of communication method on the use of DMI. Does the use of DMI vary as a function of whether it is provided to users or whether they have to seek it out? Fifthly, are there differences in DMI use between countries? As described, posing the interview question neutrally would better fit with different international DMI approaches to providing or offering citizens this information. Sixthly, what are peoples’ actual experiences with DMI? In this study, participants imagined these different types of DMI and their perceived advantages and disadvantages. However, these may not reflect their actual use. Finally, to the best of our knowledge there is no evidence on the impact of paper compared to digital medium on use of this information. Thus, another research question would be is the traditional paper WMI approach more effective in ensuring people read this information than any or all formats of DMI? Perhaps there is case to be made for continuing the paper process, if people are more likely to read it.

## 5. Conclusions

This study was an important preliminary exploration into the opinions of people regarding different options for communicating DMI. DMI are already available as websites and mobile apps. The findings from this study suggest that there are a many perceived advantages and disadvantages associated with distributing DMI and they vary between formats (e.g., websites, mobile apps, email). This sample of Canadian University students were most supportive of receiving DMI by email so it would be readily available wherever they were, because they could access it using their smartphones and they knew it was coming from a trusted source. Some reported that they would be more likely to use it because they received it rather than having to look for it. Participants also considered emailed DMI easy to access because they would not need to remember additional login information and easy to search and locate specific information. They also appreciated the flexibility of sharing or printing DMI if it came in an email. Participants wanted it to be personalized to them specifically. Some considered emailed DMI most suitable because they would infrequently need to refer to the information and because of this they would not want an app taking up storage space on their phone. The results on findability or how easy the DMI would be to locate were mixed. Some participants believed it would be easy to search their inbox and locate the DMI when they needed it and others thought it would be inconvenient. The possible security risks of communicating this information using email was also identified as a possible weakness of this format. Despite email being identified as the most ideal DMI format in this study, it is currently not being used to provide prescription medication information to citizens. Studies have found that use rates of medication information for citizens are typically low (e.g., [35]). However, a factor in these low usage rates may be due to the medium by which they are communicated. That is, if we are not aligning the way we deliver this information with citizens’ preferences and the opportunities inherent to DMI, people may be less compelled to use these materials.

The findings from this study are important in that they suggest that the formats we use for deploying information about prescription medications, as well as factors associated with these formats, are important and may influence users’ opinions and use of these materials. This initial exploration into DMI preferences with Canadian University students was valuable. However, given the dearth of evidence regarding DMI, more studies are warranted to explore preferences in more depth as well as other various topics related to DMI (e.g., usage rates, experiences, design, content) to ensure we are optimizing DMI deployment and thereby maximizing its use to support safe medication taking practices.

## Figures and Tables

**Table 1:** Summary of which themes were reported as advantages and/or disadvantages for email, mobile applications (apps), and online digital medication information (DMI). Legend: light grey rectangles + = at least one reported advantage, dark grey rectangles (−) = at least one reported disadvantage, and medium grey rectangles ± = at least one reported advantage and one disadvantage. Note: Other DMI formats may also exhibit these advantages and disadvantages, but only those that participants reported during the semi-structured interviews are indicated.

Theme	Advantage (+) or Disadvantage (−) Reported for		
	Email	Mobile App	Online
Availability	+	+	+
Ease of Access	+	(−)	±
Findability	±		±
Communication Method	+	+	(−)
Storage	+	(−)	
Security	(−)		+
Flexibility	+		
Personalization	+		
Searchability	+		
Frequency of Use	+	±	
Trustworthiness	+		+
Comprehensiveness		+	+
Layered Content		+	
Helpful in an Emergency		+	
Medication Reminders		+	

**Table 2:** Quotes illustrating the themes of reported advantages and disadvantages identified for email digital medication information (DMI). + = a reported advantage, (−) = a reported disadvantage, and ± = both a reported advantage and disadvantage. Note: Only those themes identified for email DMI were included in this table.

Theme	+ or (−)	Illustrating Quote
Availability	+	Participant 1: “And just having it on my email, then I can access it wherever, whenever.”
Ease of Access	+	Participant 1: “I think in an email, where it’s like, I don’t have to make extra accounts for logging into a patient portal or anything like that.”
Findability	±	Participant 9: “I can keep a digital version on email whenever I need it, I can find it.” Participant 3: “Because I find emails—you have to go through your emails, and search.”
Communication Method	+	Participant 15: “Email would be good because it would be straight to me … an app, you have to actually go and get it, or a website database … it’s not like it’s coming to you, you have to go to it, and I don’t think I would do that yet.” (push communication) Participant 7: “If I knew that I was able to respond to that email and be like, “I’m having this reaction. Should I go to the doctor?” Or, “Is this normal?” … as opposed to going into a pharmacy or having to go back to a walk-in clinic.” (interactive communication)
Storage	+	Participant 17: “Emails I can keep forever.”
Security	(−)	Participant 8: “If you’re emailing things to people it’s not really secure, and then people can potentially find out what kind of medications you’re taking and all that.”
Flexibility	+	Participant 13: “If it’s on your email, then you could potentially print it out. You could back it up. You could save it to a Google drive. You could do a lot of things with it.”
Personalization	+	Participant 7: “Email … I feel like if it was an app or a website, it would get right back to that really medically, jargony, generic information. Whereas if there would be a way to get the instructions that were more specific and simplified to me, I would prefer that.”
Searchability	+	Participant 2: “I can also search in it, if it’s a PDF, for specific things so it’s easier to find what I’m looking for.”
Frequency of Use	+	Participant 22: “Email because I get all my emails to my phone … every time I get an email, I’m really good about checking them right away … I would read through that.”
Trustworthiness	+	Participant 21: “That would be nice because then I would know that’s a valid resource.”

**Table 3:** Quotes illustrating the themes of reported advantages and disadvantages identified for mobile app digital medication information (DMI). + = a reported advantage and (−) = a reported disadvantage. Note: Only those themes identified for mobile app DMI were included in this table.

Theme	+ or (−)	Illustrating Quote
Availability	+	Participant 23: “It’s nice when they give you a sheet but obviously, you’re prone to losing it so it become a nice to be able to have a backup copy, like if you could access it through that app.”
Ease of Access	(−)	Participant 25: “I feel like the app would just be clumsy and a lot of steps to have to go through to get to the thing.”
Communication Method	+	Participant 18: “I think the mobile app would be … ideal if my physician or family practice … or even the pharmacy. If they had their own personalized application that they had their patients sign up on, that would be great. So some sort of portal that connects the patient to the provider absolutely.”
Storage	(−)	Participant 13: “I wouldn’t like the idea of having to have a specific app that takes up space on my phone, just so that I could look up these things.”
Frequency of Use	+	Participant 9: “I’d use the app more often.”
	(−)	Participant 35: “I think an app wouldn’t necessarily be that practical for me. Like I said, I don’t have very many prescriptions.”
Comprehensiveness	+	Participant 3: “It would give me my need, like, ‘I’m taking this. Oh, I’d better refill this. It’s time to get some more.’”
Layered Content	+	Participant 27: “That could have a simplified version … it can be a bunch of text and pictures and stuff, and you can click the button and it simplifies everything. You can always have something like that, that’s just like, ‘Here’s the basic points,’ and then another tab that’s like, ‘Here’s the really detailed information.’ So, I think an app would be kind of cool.”
Helpful in an Emergency	+	Participant 10: “I think that a key feature for that actually might be that the app would push a link directly to a phone’s home screen. So, if someone needed to respond to an emergency, like an outside person, they could find this phone and then they’d have access to this information without knowing the password of this individual’s phone.”
Medication Reminders	+	Participant 16: “Maybe if I had something chronic that I did need reminders, and it was a little bit more encompassing, like I don’t know, diabetes or something, where I needed to do the tests and it was a bit more complicated, maybe an app would be useful.”

**Table 4:** Quotes illustrating the themes of reported advantages and disadvantages identified for online digital medication information (DMI). + = a reported advantage and (−) = a reported disadvantage. Note: Only those themes identified for email DMI were included in this table.

Theme	+ or −	Illustrating Quote
Availability	+	Participant 3: “Online, that would be great. Because then, I would be able to access it whenever I needed to.”
Ease of Access	+	Participant 25: “Just a link on the website or like—on the London Drugs [Canadian pharmacy chain] website would work or on the manufacturer’s website or just somewhere.”
	(−)	Participant 13: “I wouldn’t like the idea that the only way for me to get my prescription medication [information] would be for me to make an account with a username and a password that I have to remember.”
Findability	+	Participant 6: “If there was a master website that you could always you could just Google and find out, then that would be nice.”
	(−)	Participant 13: “I have to remember the website.”
Communication Method	(−)	Participant 2: “If it was just found online, I probably wouldn’t take it upon myself to go look for it.”
Security	+	Participant 8: “I think if there was a database that you could look up, or some kind of secure access point, just because—I don’t really care, personally, but I would be concerned about confidentiality for other people.”
Trustworthiness	+	Participant 32: “online format that I knew was reliable, because it was from my pharmacy or Health Canada or something.”
Layered Content	+	Participant 32: “I think if it were just a website that had a directory of all the medications would be useful.”
